# Standard diagnostics with and without urine-based lipoarabinomannan testing for tuberculosis disease in HIV-infected patients in a high-burden setting–A cost-effectiveness analysis

**DOI:** 10.1371/journal.pone.0288605

**Published:** 2023-07-14

**Authors:** Ginenus Fekadu, Yingcheng Wang, Joyce H. S. You

**Affiliations:** School of Pharmacy, Faculty of Medicine, The Chinese University of Hong Kong, Hong Kong, SAR, China; Kings College Hospital, UNITED KINGDOM

## Abstract

**Background:**

Recent clinical findings reported the reduced mortality associated with treatment guided by sputum-based molecular test with urine-based lipoarabinomannan (LAM) assay for tuberculosis (TB) disease in HIV-infected individuals. We aimed to evaluate the cost-effectiveness of sputum-based Xpert tests with and without urine-based LAM assays among HIV-infected individuals with signs and symptoms of TB disease (TBD) from the perspective of South African healthcare providers.

**Methods:**

A one-year decision-analytic model was constructed to simulate TB-related outcomes of 7 strategies: Sputum smear microscope (SSM), Xpert, Xpert Ultra, Xpert with AlereLAM, Xpert Ultra with AlereLAM, Xpert with FujiLAM, and Xpert Ultra with FujiLAM, in a hypothetical cohort of adult HIV-infected individuals with signs and symptoms of TB. The model outcomes were TB-related direct medical cost, mortality, early treatment, disability-adjusted life-years (DALYs) and incremental cost per DALY averted (ICER). The model inputs were retrieved from literature and public data. Base-case analysis and sensitivity analysis were conducted.

**Results:**

In the base-case analysis, the Xpert Ultra with FujiLAM strategy showed the highest incidence of early treatment (267.7 per 1000 tested) and lowest mortality (29.0 per 1000 tested), with ICER = 676.9 USD/DALY averted. Probabilistic sensitivity analysis of 10,000 Monte Carlo simulations showed the cost-effective probability of Xpert Ultra with FujiLAM was the highest of all 7 strategies at the willingness-to-pay (WTP) threshold >202USD/DALY averted.

**Conclusion:**

Standard sputum-based TB diagnostic Xpert Ultra with urine-based FujiLAM for TBD testing in HIV-infected individuals appears to be the preferred cost-effective strategy from the perspective of the health service provider of South Africa.

## Introduction

Tuberculosis (TB), caused by *Mycobacterium tuberculosis* (MTB), is the leading cause of death in HIV-infected people worldwide. Over 187,000 fatalities were reported in 2021, accounting for roughly one-third of AIDS-related mortality [1]. South Africa is a country with a high HIV-TB burden, and the incidences of TB and HIV-positive TB were 513 and 274 per 100,000 population, respectively [1]. The disease burden of HIV-associated TB in low- and middle-income countries is high, resulting from missed/late diagnosis of TB disease (TBD) and consequential late-treatment-associated mortality [1, 2].

Sputum smear microscopy (SSM), the identification of acid-fast bacilli through microscopic examination of stained sputum smears, has been the cornerstone of TBD diagnosis. SSM has good specificity (>90%), yet the sensitivity was low (<70%) with a limit detection of 5000–10,000 bacilli per milliliter of sputum [3–5]. In 2011, South Africa made a policy decision to replace SSM with Xpert MTB/RIF as the initial diagnostic test for TB across the entire national laboratory service [6]. World Health Organization (WHO) also recommended replacing SSM as the initial diagnostic test with rapid newer diagnostic technologies [2]. Nonetheless, SSM remains necessary to monitor response to treatment [2, 7].

Xpert MTB/RIF (Cepheid, Sunnyvale, USA) (Xpert), is an automated molecular polymerase chain reaction (PCR) assay for the detection of MTB-complex [7, 8]. The sputum-based Xpert assay detects MTB and rifampicin (RIF) resistance within 2 hours [7]. The Xpert MTB/RIF Ultra (Cepheid, Sunnyvale, USA) assay (Xpert Ultra), is the next-generation Xpert with higher sensitivity (87.6%) than Xpert (70.7%) in HIV-infected patients [2, 9].

The sputum-based TB testing in HIV-infected patients remains a critical challenge because of the difficulty to produce required sputum and low organism loads of MTB-complex in sputum [1, 2, 10]. Lateral flow urine lipoarabinomannan assay is an immunochromatographic biomarker test for antigen detection of mycobacterial lipoarabinomannan (LAM), glycolipid found in the outer cell wall of Mycobacteria [11, 12]. Alere Determine TB LAM Ag (Alere Determine TB LAM Ag, Abbott, San Diego, USA) (AlereLAM), is the first-generation Lateral flow urine lipoarabinomannan test with a quick turnaround time (25 minutes). Fujifilm SILVAMP TB-LAM (FujiLAM; Fujifilm, Tokyo, Japan) (FujiLAM) is another urine assay (turnaround time < 1 hour) with higher sensitivity (70.7%) than AlereLAM (34.9%) for MTB detection in HIV-infected patients [13, 14]. The lateral flow urine lipoarabinomannan assays enhance the logistical efficiency of MTB diagnostics, including its ease of use, inexpensive, rapid turnaround time, and readiness to be performed at the point-of-care [7, 10, 15]. Adding the lateral flow urine lipoarabinomannan assays to the standard diagnostics of MTB significantly improve TBD diagnosis and reduce mortality. A pragmatic, multicounty, randomised trial (n = 2528) reported that the addition of AlereLAM to standard diagnostics was associated with a relative risk reduction of 17% (95% CI: 4–28%) in mortality at 8 week in HIV-positive patients [11]. A Cochrane review (n = 2 trials with 5102 participants) further reported that adding the AlereLAM test to the routine TB diagnostic testing reduced the risk of mortality by 15% (RR: 0.85, 95% CI: 0.76–0.94) at 8 weeks in HIV-positive patients [13].

No previous studies had evaluated the cost-effectiveness of sputum-based Xpert systems with or without urine-based new generation LAM assays for TBD testing among HIV-infected individuals in high-burden settings. To inform the health policy and service provider on the outcomes of adding the lateral flow urine lipoarabinomannan assays to standard Xpert testings for MTB in HIV-positive patients, we aimed to evaluate the potential cost-effectiveness of sputum-based Xpert tests with and without urine antigen assays for LAM among HIV-infected individuals with signs and symptoms of TB from the perspective of South African healthcare providers.

## Methods

### Model design

**Fig 1:**
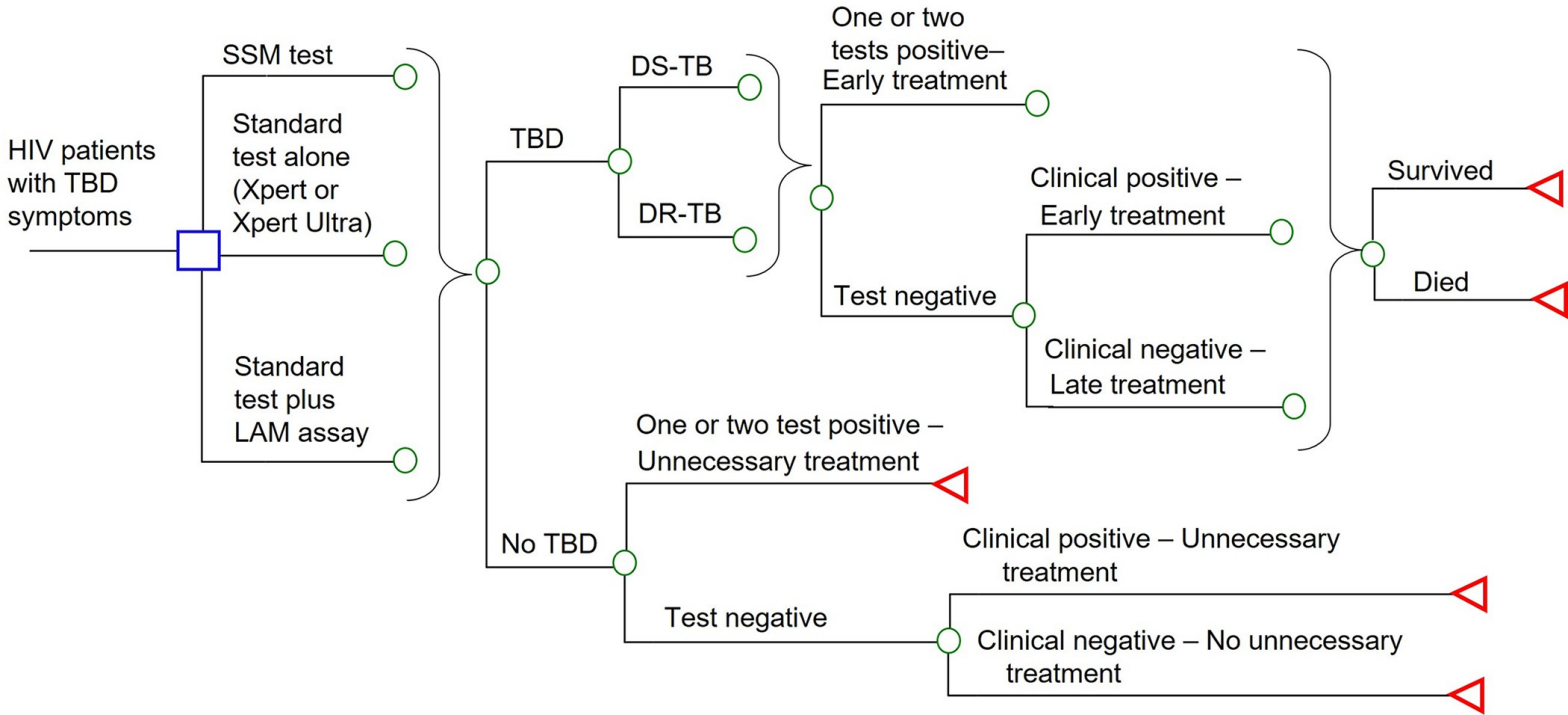
Simplified decision-tree for TB testing in HIV-infected individuals with signs and symptoms of TBD. DR-TB: drug-resistant tuberculosis; DS-TB: drug susceptible tuberculosis; LAM: mycobacterial lipoarabinomannan; SSM: sputum smear microscope; TBD: tuberculosis disease.

A decision-analytic model (**Fig 1**) was constructed to simulate the potential TB-related outcomes of a hypothetical cohort of adult HIV-infected individuals with signs and symptoms of TBD. Decision-analytic modelling is a well-accepted tool, by incorporating evidence-based probabilities of clinical events, disease-specific utility and cost parameters, for assessment of health technology. The health economic evidence generated by decision-analytic modelling facilitate health policy and decision makers on informed decision of resources allocation [16, 17].

Seven diagnostic strategies were evaluated: (1) SSM, (2) Xpert, (3) Xpert Ultra, (4) Xpert with AlereLAM, (5) Xpert Ultra with AlereLAM, (6) Xpert with FujiLAM, and (7) Xpert Ultra with FujiLAM. A one-year model time horizon was employed to allow adequate time to capture the prominent clinical and economic outcomes of testing and TB treatment. The model outcomes were TB-related direct medical cost, number of TB patients who received early treatment, mortality, and disability-adjusted life-years (DALYs).

At the model entry of all testing strategies, the hypothetical individuals might (or might not) be infected with TBD. Those who had TBD might be infected by RIF-resistant or RIF-sensitive MTB. The TBD patients would receive early anti-TB treatment if the diagnostic test(s) or clinical judgment was positive for TBD. Early anti-TB treatment was defined as therapy started during the initial assessment. It was considered as a late treatment when diagnostic test(s) and clinical judgement were all false negative, and the treatment was initiated after positive sputum culture results had been reported (usual sputum culture turn-around-time was 4–8 weeks). The TBD patients, with early or late treatment, might survive or die of TBD. Individuals without TBD but received early anti-TB treatment unnecessarily (as indicated by false positive results of diagnostic assay(s) or false positive clinical judgement-based diagnosis) would receive anti-TB treatment until TBD was ruled out by negative sputum culture results.

For the SSM (in the absence of Xpert testings) strategy, the patients would be tested by microscope smear with two consecutive sputum samples. Those who were tested MTB positive would receive early drug-susceptible TB (DS-TB) treatment with a standard first-line regimen (rifampin, isoniazid, pyrazinamide, and ethambutol for a two-month intensive phase followed by a four-month continuation phase of rifampin and isoniazid) [18, 19].

For the Xpert and Xpert Ultra (standard testing alone) strategies, the patients would be tested by Xpert or Xpert Ultra on a single sputum sample. Those who were tested MTB positive would receive early TB treatment. Patients with negative RIF-resistance (per Xpert or Xpert Ultra) were managed as DS-TB (as described above). Patients with Xpert or Xpert Ultra positive for RIF resistance were treated as drug-resistant tuberculosis (DR-TB) by second-line anti-TB agents in accordance with WHO and South African national TB guidelines [20, 21]. The second line anti-TB treatment consisted of bedaquiline (used for 6 months), in combination with levofloxacin/moxifloxacin, ethionamide, isoniazid (high-dose), ethambutol, clofazimine and pyrazinamide for 4 months (with the possibility of extending to 6 months if the patient remains sputum smear positive at the end of 4 months); followed by 5 months of treatment with levofloxacin/moxifloxacin, ethambutol, clofazimine, and pyrazinamide [20, 21].

For the four-standard testing plus LAM assay strategies (Xpert with AlereLAM, Xpert Ultra with AlereLAM, Xpert with FujiLAM, and Xpert Ultra with FujiLAM), individuals were tested by single-sample urine-based LAM assay and single-sample sputum-based Xpert/Xpert Ultra assay. Patients with either or both of the tests positive for MTB would receive early TB treatment. The selection of DS-TB or DR-TB regimens was guided by the Xpert/Xpert Ultra RIF resistance testing results (as described above).

Patients with negative results in the SSM strategy, negative Xpert/Xpert Ultra in the two standard testing alone strategies, and those with negative testing results by both assays in the four standard testing plus LAM assay strategies were further evaluated clinically by (but not limited to) chest radiography, diagnostic oral antibiotic trial [7], using first-line empirical treatment (broad spectrum beta-lactams and macrolides) according to South African guideline for the management of pneumonia [22]. Individuals with a positive clinical judgement-based diagnosis of TBD would receive early first-line anti-TB regimen. In all study arms, mycobacterial culture and (if culture was positive) drug susceptibility testing would be conducted on sputum specimen. Patients with TBD who did not receive early anti-TB treatment would receive late treatment when positive sputum culture of MTB was reported. The treatment regimen would be modified when drug susceptibility results were available.

### Clinical inputs

All model inputs are shown in **Table 1**. A search for clinical probabilities was performed in Medline (covering period 2000–2022), public data of WHO, and Department of Health of South Africa to estimate model parameters using keywords “tuberculosis disease”; “active tuberculosis”; “SSM”, “Xpert”; “Xpert Ultra”; “AlereLAM”; “FujiLAM”; and “HIV”. The selection criteria for clinical studies were: (1) Reports written in English; (2) adult HIV-infected patients, and (3) TB testing results and/or mortality were reported. Meta-analyses or randomized controlled trials were preferred sources for the model inputs. A study was included if the data pertaining to the model inputs were available. The weighted average was used to estimate the base-case value if multiple sources were obtained, whilst the upper and lower limits formed the range for sensitivity analysis.

**Table 1:** Model input parameters.

Parameters	Base-case value	Range for sensitivity analysis	Distribution	Reference
***Clinical inputs***				
TBD prevalence in HIV-infected patients	27.46%	21.97%–32.95%	Beta	[23]
Proportion of DR-TB in TBD cases	7.12%	3.10%–8.54%	Beta	[24]
Sensitivity for TBD in HIV-infected patients				
SSM	61.79%	54.90% –68.40%	Beta	[25]
AlereLAM	34.90%	19.50%–50.90%	Beta	[26]
FujiLAM	70.70%	59.00%–80.80%	Beta	[26]
Xpert for MTB detection	74.90%	58.70%–86.20%	Beta	[9]
Xpert Ultra for MTB detection	87.60%	75.40%–94.10%	Beta	[9]
Xpert for RIF resistance	95.30%	90.00%–98.10%	Beta	[9]
Xpert Ultra for RIF resistance	94.90%	88.90%–97.90%	Beta	[9]
Clinical (empiric) diagnosis for test negative	61.00%	55.00%–67.00%	Beta	[27]
Specificity for TBD in HIV-infected patients				
SSM	99.73%	99.60%–99.80%	Beta	[25]
AlereLAM	95.30%	92.20%–97.70%	Beta	[26]
FujiLAM	90.90%	87.20%–93.70%	Beta	[26]
Xpert for MTB detection	99.70%	98.60%–100.00%	Beta	[9]
Xpert Ultra for MTB detection	92.80%	82.30%–97.00%	Beta	[9]
Xpert for RIF resistance	98.80%	97.20%–99.60%	Beta	[9]
Xpert Ultra for RIF resistance	99.10%	97.70%–99.80%	Beta	[9]
Clinical (empiric) diagnosis for test negative	69.00%	66.00%–72.00%	Beta	[27]
Mortality rate among DS-TB in HIV-infected patients	9.63%	6.37%–20.24%	Beta	[28]
Mortality rate among DR-TB in HIV-infected patients	21.00%	13.00%–42.63%	Beta	[29]
Hazard ratio of mortality with late TB treatment	1.36	1.07–1.77	Lognormal	[30]
***Utility inputs***				
Utility				
DS-TB treatment	0.69	0.57–0.77	Triangular	[31]
DR-TB treatment	0.51	0.39–0.73	Triangular	[31]
Age-specific utility				[32]
18–65 years	0.92	—		
>65 years	0.84	—		
HIV patient age (years)	35	30–42	Triangular	[14]
***Cost inputs* (USD)**				
Cost per test				
SSM	10	8–12	Gamma	[33, 34]
AlereLAM	4	3–5	Gamma	[35]
FujiLAM	8	4–19	Gamma	[36]
Xpert	26	21–31	Gamma	[37]
Xpert Ultra	26	21–31	Gamma	[35, 37]
Mycobacterial culture	25	20–30	Gamma	[38]
Drug susceptibility testing	63	50–76	Gamma	[34]
Chest X-ray	39	31–47	Gamma	[33]
TB outpatient clinic visit (cost per visit)	38	30–46	Gamma	[38]
Oral antibiotics trial (treatment course)	7	6–8	Gamma	[39]
Cost per case				
DS-TB treatment	461	369–553	Gamma	[38]
DR-TB treatment	12,161	9,729–14,593	Gamma	[38]
TB-related mortality	4,698	3,758–5,638	Gamma	[40]

DR-TB: drug resistant tuberculosis; DS-TB: drug susceptible tuberculosis; HIV: human immunodeficiency virus; MTB: *mycobacterial tuberculosis*; RIF: rifampicin; SSM: sputum smear microscope; TB: tuberculosis; TBD: tuberculosis disease.

The TBD prevalence (27.46%) in HIV-infected patients was retrieved from findings of a 8-year prospective study (n = 1544) following patients receiving antiretroviral therapy in a community-based cohort in South Africa [23]. The annual report (2021/2022) of the Department of Health of South Africa indicated that the prevalence of DR-TB among TBD cases in South Africa was 7.12% [24].

The sensitivity and specificity of SSM for the diagnosis of HIV-associated pulmonary TB was retrieved from a diagnostic accuracy study (n = 2216 subjects) in a high-burden setting [25]. The sensitivity and specificity inputs of AlereLAM and FujiLAM for TBD were retrieved from the results of a meta-analysis of diagnostic accuracy of LAM assay among people living with HIV (n = 5 prospective cohort studies with 1,595 patients) [26]. The model parameters on sensitivity and specificity of Xpert and Xpert Ultra were obtained from the findings of a systematic review (n = 9 studies with 3,500 participants) compared the diagnostic accuracy of Xpert and Xpert Ultra for the detection of MTB and RIF resistance in adults with presumptive pulmonary TB (including subgroup analysis among HIV-infected patients) [9]. The accuracy of clinical judgement-based diagnosis for test-negative individuals was estimated from findings of a meta-analysis (n = 24 studies) comparing the accuracy of Xpert, microscopic observation drug susceptibility assay, and the WHO algorithm for diagnosis of smear-negative TBD [27].

The mortality rate among HIV-infected patients with DS-TB (9.63%) was retrieved from a retrospective 8-year data analysis (n = 2,551,058) of TBD treatment with the data of national tuberculosis treatment register in South Africa [28]. A systematic review and meta-analysis (n = 38 studies with 9,279 patients) of short regimens with bedaquiline and linezolid-based treatment outcomes in DR-TB patients with HIV reported pooled mortality rate to be 21.00% [29].The hazard ratio of mortality associated with late versus early TBD treatment (1.36;95%CI: 1.07–1.77) was reported in a multicentre prospective cohort study (n = 740) evaluating the delayed diagnosis of TBD and its effect on mortality in persons living with HIV in Eastern Europe [30].

### Health utility inputs

The effectiveness of each testing strategy was evaluated in terms of the TB-related DALY, a time-based measure that combines the years of healthy life lost due to morbidity-related disability (people living in a health state of less than good health) and years of healthy life lost due to premature mortality [41, 42]. WHO used the DALY to estimate the global burden of disease [42]. Indeed, this approach helps to get a better estimate of a population that may have a high mortality rate but tends to not describe their health as poor quality [43–45]. It also allows experts and policymakers to take a large-scale evaluation of a particular disease burden of the population with an average life expectancy, and make recommendations to improve accordingly [43, 44]. The expected DALYs of the TB-related morbidity were approximated using the patient time spent in the TB disease state and loss of utility related to the TB disease state. The utility loss was estimated by the difference between the corresponding TB disease state’s utility and the age-specific utility of healthy individuals [41, 42]. DALYs resulting from TB-related mortality were approximated by the age-specific health utilities and loss of life-years (age-specific remaining life expectancy derived from the South African life expectancy tables). The base-case age of the HIV-infected individuals (35 years) was retrieved from the demographic findings of a diagnostic accuracy study of TBD testing among HIV-infected individuals in South Africa [14]. The life expectation of a 35-year-old was 36 years in South Africa [46]. The age-specific health utility values (0.92 for ages 18–65 years and 0.84 for ages >65 years) were derived from the health-related quality of life study on medical conditions using population measures [32].

The utility values of DS-TB (0.69) and MDR-TB (0.51) disease treatment were retrieved from the findings of a health-related quality-of-life measures study in Thailand TB patients [31]. In the absence of local data, it is acceptable to transfer and adopt the health state valuations (utilities) from other settings after adjustment for appropriate national value sets (like age-specific life expectancy) [47–49]. The expected DALYs for TB-related mortality were discounted to the year 2023 with an annual rate of 3%.

### Cost inputs

The cost analysis was conducted from the perspective of healthcare provider in South Africa. The cost items included diagnostic tests (SSM, Xpert, Xpert Ultra, LAM assays, culture, drug-susceptibility test, chest X-ray, diagnostic antibiotic trial), outpatient care, DS-TB treatment, DR-TB treatment, and TB-related mortality. Cost parameters were derived from literature and publicly available data (**Table 1**).

The cost of AlereLAM was estimated from the diagnostic and health products catalog of the Stop TB Partnership [35]. The cost of urine FujiLAM was not published, and the cost per test adopted the cost parameter of a health economic evaluation of LAM assay for TB detection [36]. The cost of Xpert and Xpert ultra tests were estimated from the diagnostic and health products catalog of the Stop TB Partnership, and laboratory cost analysis of rapid molecular assays for TB diagnosis in South Africa [35, 37]. Costs of SSM, Mycobacterial culture, drug-susceptibility test, and chest X-ray were reported by a cost-analysis of diagnosis and management of TB in South Africa [33, 34, 38]. The cost of oral antibiotics trial using the first-line empirical treatment for pneumonia was estimated from the supply and delivery of anti-infective medicines of the Department of Health of South Africa [39].

The cost of TB outpatient clinic visits and treatment cost per case of DS-TB and MDR-TB were derived from the results of a cost analysis of TB diagnosis and management in South Africa [38]. The cost of TB-related mortality was obtained from a published study of costs and processes of in-patient TB management in South Africa [40]. All costs were adjusted to 2023 USD values (USD1.00 = South African Rand (ZAR) 17.00) using the South African Consumer Price Index (CPI) for health services (if applicable) [50].

### Cost-effectiveness, sensitivity and scenario analyses

The analysis was performed using TreeAge Pro 2022 (TreeAge Software Inc, Williamstown, MA, USA) and Excel 365 (Microsoft Corporation, Redmond, WA, USA). All strategies were compared stepwise to the next less costly strategy. The incremental cost per DALY averted (ICER) of each strategy, compared to the next less costly strategy, was calculated using the equation: ICER = ΔCost/ΔDALYs.

A strategy was dominated when it resulted in either (1) higher DALYs at a higher cost, or (2) ICER higher than that of a more effective strategy. The dominated strategies were eliminated from further cost-effectiveness analysis. After the elimination of the dominated strategies, ICER of each remaining strategy, compared to the next less costly strategy, was presented. A strategy was accepted as the preferred cost-effective option if it resulted in (1) lower DALYs at a lower cost, or (2) lower DALYs at a higher cost, and the ICER was less than the willingness-to-pay (WTP) threshold. According to the WHO recommendations, a healthcare intervention is considered highly cost-effective when the ICER is less than the gross domestic product (GDP) per capita [51]. The GDP per capita of South Africa (USD7,055 in 2021) [52] was adopted as the WTP threshold in the base-case analysis.

All parameters were examined by the one-way sensitivity analysis across the ranges specified in **Table 1**. The range for sensitivity analysis was either the 95% confidence interval or high/low values of the model input, if available. If both the 95% confidence interval and high/low values were not reported, ± 20% of the base-case value was used to form the range for sensitivity analysis. The probabilistic sensitivity analysis was performed using Monte Carlo simulation. The cost and DALYs of each testing strategy were recalculated in 10,000 iterations, by randomly drawing all model input values simultaneously from the parameter-specific probability distribution. The probabilities of each strategy to be accepted as cost-effective were examined over a wide range of WTP thresholds from 0 to 14,110 (2× GDP per capita) USD/DALY by the acceptability curves with the cost-effectiveness acceptability frontier.

It was reported that the sensitivity and specificity of urine based LF-LAM tests vary in different groups of HIV-infected individuals [2, 26]. A scenario analysis on the same 7 testing strategies was conducted for 5 subgroups of HIV-infected individuals: Inpatient status, outpatient status, CD4 count ≤ 100 cells/μL, 100–200 cells/μL and >200 cells/μL. The model inputs for the sensitivity and specificity of the subgroup analyses (**S1 Table**) were derived from the same meta-analysis on diagnostic accuracy of LAM assay among people living with HIV described above [26].

## Results

### Base-case analysis

The base-case results are shown in **Table 2**. Of the 7 strategies examined, 4 strategies were dominated, and eliminated from further cost-effectiveness analysis. Three strategies remained in the cost-effectiveness analysis were SSM, Xpert Ultra, and Xpert Ultra with FujiLAM. Compared with the SSM, the Xpert Ultra averted 0.0448 DALYs at a higher cost of USD3.2 (ICER = 71.4 USD/DALY averted). Compared with the Xpert Ultra strategy, Xpert Ultra with FujiLAM averted 0.0065 DALYs at an incremental cost of USD4.4 (ICER = 676.9). The Xpert Ultra with FujiLAM generated the lowest DALYs with ICER (676.9 USD/DALY averted) less than the WTP threshold (7,055 USD/DALY), and was therefore the preferred cost-effective option.

**Table 2:** Base-case results.

Testing strategy	TBD patients received early treatment^†^	TB-related mortality^†^		Direct cost (USD)		Incremental cost (USD)		DALYs	DALY averted		ICER
SSM	217.0	31.5		691.0		-		0.6869	-		
Xpert Ultra	259.0	29.3		694.2		3.2		0.6421	0.0448		71.4
Xpert Ultra + AlereLAM	263.3	29.2		696.4		2.2		0.6389	0.0032		687.6*
Xpert Ultra + FujiLAM	267.7	29.0		698.6		2.2		0.6356	0.0033		660.0
Xpert	244.0	30.0		700.1		1.5		0.6551	-0.0195		-79.1*
Xpert + AlereLAM	252.8	29.7		700.6		2.0		0.6486	-0.0130		-155.6*
Xpert + FujiLAM	261.7	29.3		701.0		2.4		0.6420	-0.0064		-380.9*
Excluding dominated strategies											
	Total direct cost (USD)		Incremental cost (USD)		DALYs		DALY averted			ICER	
SSM	217.0		-		0.6869		-			-	
Xpert Ultra	694.2		3.2		0.6421		0.0448			71.4	
Xpert Ultra + FujiLAM	698.6		4.4		0.6356		0.0065			676.9	

^†^per 1000 individuals tested

* Dominated strategies

DALY: disability-adjusted life years; SSM: sputum smear microscope; TBD: tuberculosis disease; TB: tuberculosis

ICER = incremental cost/DALY averted (comparing to the next less costly strategy)

### One-way sensitivity analysis

**Fig 2:**
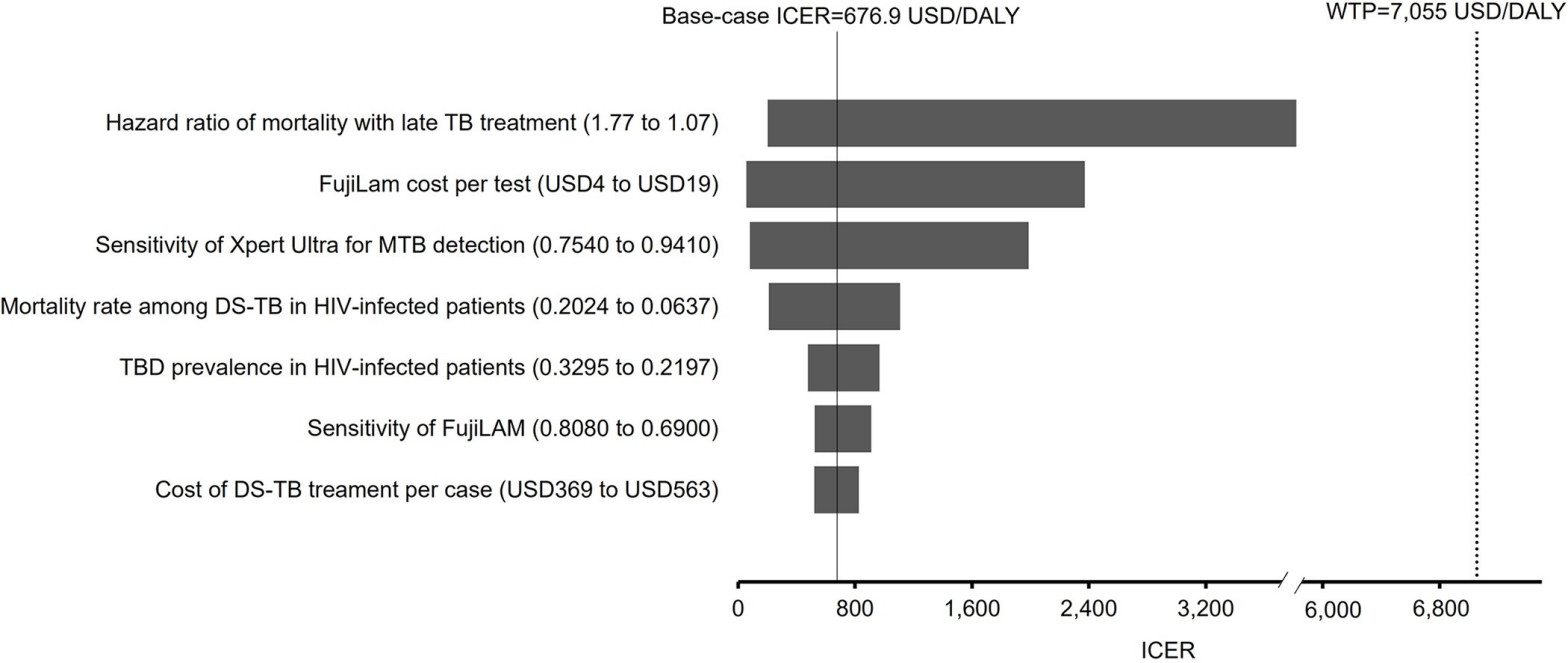
Tornado diagram of influential parameters on the ICER of FujiLAM + Xpert Ultra versus Xpert Ultra identified in a one-way sensitivity analysis. DALY: disability-adjusted life years; ICER: incremental cost-effectiveness ratio; DS-TB: drug susceptible tuberculosis; MTB: *Mycobacterium tuberculosis;* TB: tuberculosis; TBD: tuberculosis disease; WTP: willingness-to-pay.

In one-way sensitivity analysis, the base-case results were robust to the variation of all model parameters, and no threshold parameter was identified to change the base-case cost-effectiveness results. The variation of seven parameters were found to change the ICER of Xpert Ultra with FujiLAM strategy by more than 20% from the base-case include (**Fig 2**): Hazard ratio of mortality with late TB treatment, cost of FujiLAM per test, sensitivity of Xpert Ultra for MTB detection, mortality rate among DS-TB patients, TBD prevalence in HIV-infected patients, sensitivity of FujiLAM, and cost of DS-TB treatment.

To further explore the impact of sensitivity and specificity of the TB tests, extended one-way sensitivity analyses were conducted over a range of 0 to 1. **Table 3** showed the threshold values identified and corresponding change of preferred cost-effective testing strategy.

**Table 3:** Threshold values of influential parameters on the model results of a cost-effective strategy.

Variable	Base-case value	Threshold value	Change of preferred cost-effective strategy (WTP = 7,055 USD/DALY)
Sensitivity of AlereLAM	34.90%	>65.12%	AlereLAM +Xpert Ultra
Sensitivity of FujiLAM	70.70%	<40.48%	AlereLAM +Xpert Ultra
Sensitivity of Xpert for MTB detection	74.90%	>98.05%	Xpert
		87.58%-98.05%	FujiLAM + Xpert
Sensitivity of Xpert Ultra for MTB detection	87.60%	>98.05%	Xpert Ultra
		<74.94%	FujiLAM + Xpert
Sensitivity of Xpert Ultra for rifampicin resistance	94.90%	<71.10%	FujiLAM + Xpert
Specificity of Xpert Ultra for rifampicin resistance	99.10%	<90.50%	FujiLAM + Xpert

DALY: disability-adjusted life years; MTB: *Mycobacterium tuberculosis*; WTP: willingness-to-pay

### Probabilistic sensitivity analysis

Probabilistic sensitivity analysis was performed by recalculating the cost and DALYs 10,000 times with Monte Carlo simulation.

**Fig 3:**
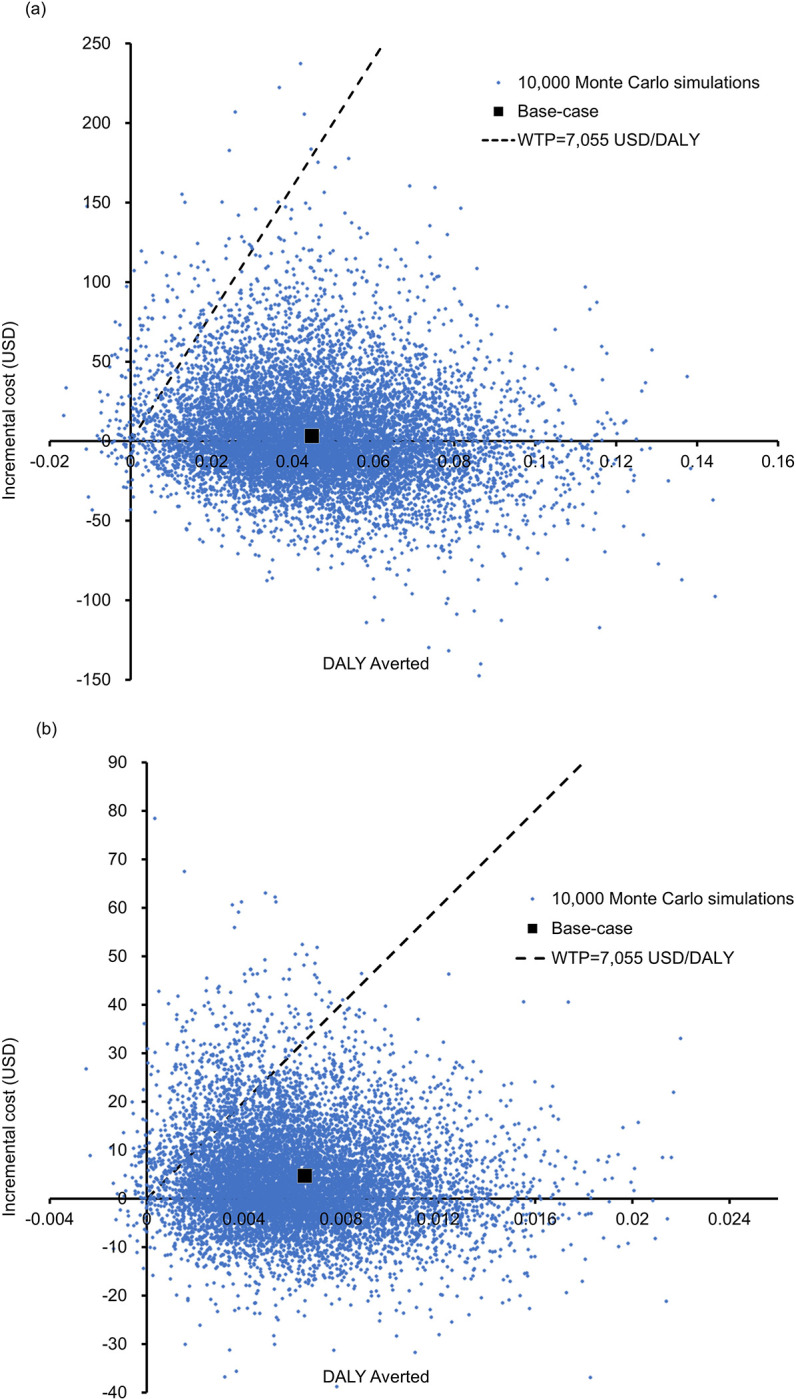
Scatter plot of the incremental cost against DALY averted by (a) Xpert Ultra versus SSM (b) Xpert Ultra with FujiLAM versus Xpert Ultra in 10,000 Monte-Carlo simulations. WTP: willingness-to-pay.

Compared with the SSM strategy, the Xpert Ultra strategy was more costly by an incremental cost of USD3.7 (95%CI: USD3.1-USD4.4; p<0.01) and averted 0.0458DALYs (95%CI: 0. 0453–0.0462; p<0.01). Of 10,000 simulations, the Xpert Ultra strategy averted DALYs (versus SSM) with the ICERs below the WTP threshold in 98.60% of the time (cost-saving in 49.16% and incurred higher cost in 49.44%) (**Fig 3A**).

Compared with the Xpert Ultra strategy, the Xpert Ultra with FujiLAM strategy showed a mean DALY reduction of 0.0065 (95%CI: 0.0061–0.0069; p<0.01), with an incremental cost of USD4.1 (95%CI: USD3.9-USD4.3; p<0.01). The incremental cost versus DALY averted by the Xpert Ultra with FujiLAM strategy was shown in a scatter plot (**Fig 3B)**. The Xpert Ultra strategy reduced DALYs at a lower cost in 36.63% of the time and averted DALYs at a higher cost with ICER<WTP threshold in 58.76% of the simulations. The Xpert Ultra with FujiLAM strategy was therefore accepted as the preferred cost-effective option in 95.39% of the 10,000 simulations.

**Fig 4:**
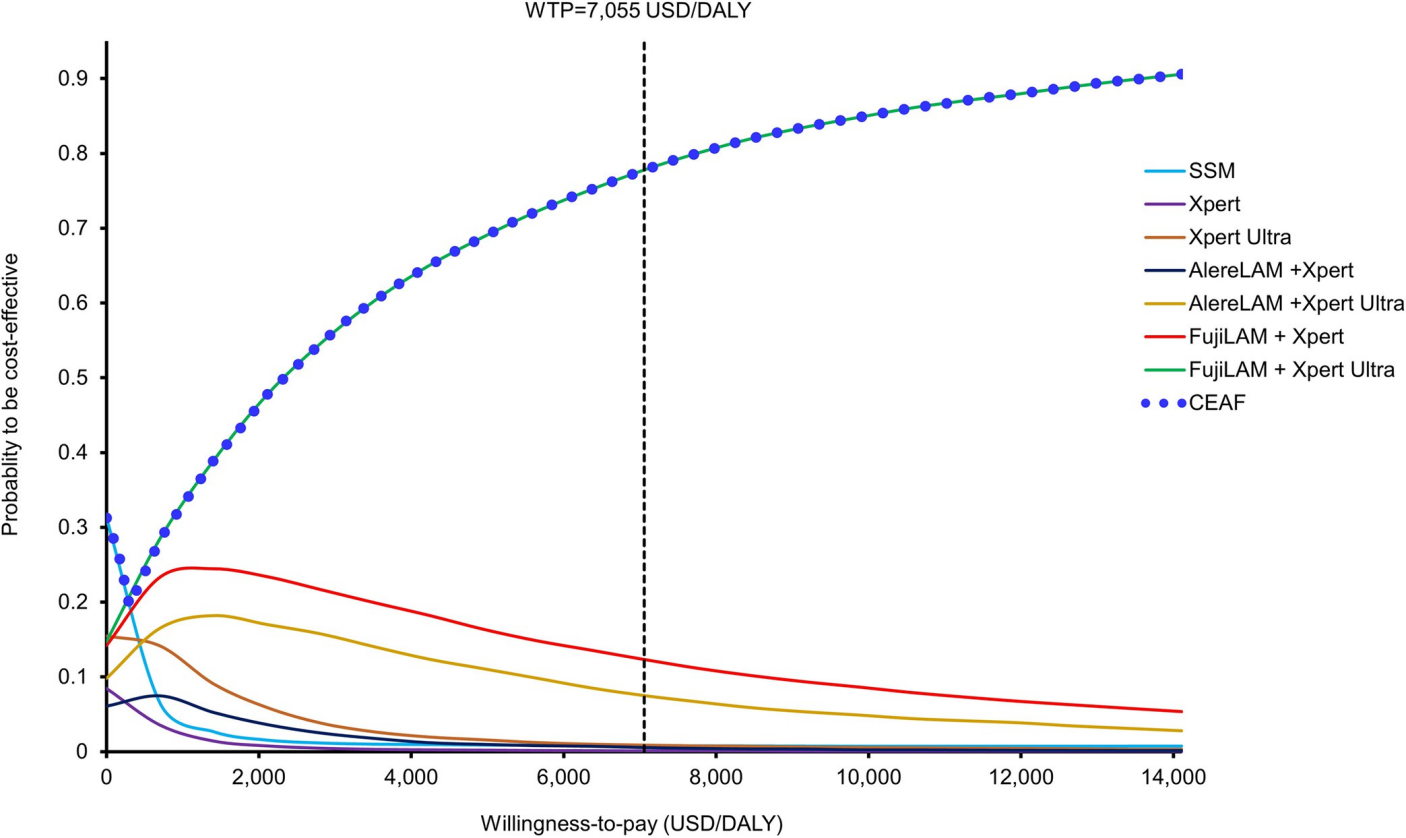
Acceptability curves and frontiers of 7 testing strategies for TB diagnosis to be cost-effective against willingness-to-pay. CEAF: cost-effectiveness acceptability frontier; WTP: willingness-to-pay.

The acceptability curves (with the cost-effectiveness acceptability frontier) presented the probability of each strategy to be cost-effective against the WTP threshold (over the range of 0–14,110 USD/DALY) (**Fig 4**). The probability of Xpert Ultra with FujiLAM to be accepted as cost-effective became the highest of all 7 strategies when the WTP threshold exceeded 202 USD/DALY, and it was 77.79% at the WTP threshold of 7,055 USD/DALY. At the WTP threshold of 2x GDP per capita (14,110 USD/DALY), the probability of Xpert Ultra with FujiLAM to be accepted as cost-effective exceeded >90%.

### Scenario analysis

The Xpert Ultra with FujiLAM remained the preferred cost-effective strategy in different HIV-infected patient statuses (inpatients and outpatients) and CD4 cell count levels. The base-case results of scenario analysis are shown in **S2 Table**.

## Discussion

The present study evaluated the cost-effectiveness of standard sputum-based Xpert/Xpert Ultra diagnostics with and without urine-based LAM assay in HIV-infected adults suspected of TBD in a high-burden country. The base-case analysis findings showed that the Xpert Ultra with FujiLAM strategy increased the likelihood of patients with TBD to start early treatment, reduced mortality, and was the preferred cost-effective option (ICER = 676.9USD/DALY) from the perspective of the South African national health service. Our scenario analysis in different patient statuses (outpatients and inpatients) and CD4 count levels (≤ 100 cells/μL, 100–200 cells/μL and >200 cells/μL) also found Xpert Ultra with FujiLAM remains the preferred cost-effective strategy.

The one-way sensitivity analysis supported the base-case findings that the cost-effectiveness of the Xpert Ultra with FujiLAM to be highly robust and no threshold value was identified throughout the variation of all model inputs. The hazard ratio of mortality associated with late TB treatment (base-case = 1.36, range; 1.07–1.77) was the most influential parameter on the ICER of FujiLAM with Xpert Ultra versus Xpert Ultra. The base-case age of patients in the present model was 35-year-old with a life expectation of 36 years (per age-specific life expectancy table of South African [46]). The DALYs resulted from the loss of expected life years of a TB patient was substantial. The variation of mortality risk associated with late treatment, therefore, showed the highest influence (of all model inputs) on the ICER. The probabilistic 10,000 Monte Carlo simulations further demonstrated the cost-effective probability of Xpert Ultra with FujiLAM was the highest of all 7 strategies when the WTP exceeded 202 USD/DALY.

The prior health economics analyses of the rapid TB testing in high-burden countries were mostly focused on the Xpert and AlereLAM. Three decision-analytic models and a microsimulation model analysis showed that the addition of AlereLAM to Xpert detected more TB cases and was cost-effective (ICER range: 24 USD/DALY-731 USD/DALY averted; 420–810 USD per years-of-life saved (YLS)) for HIV-positive patients in high-burden settings of Malawi, Uganda, South Africa, and Kenya [53–56]. A recent cost-effectiveness analysis by evaluated Xpert, Xpert with AlereLAM and Xpert with FujiLAM, and reported that ICER of Xpert+ FujiLAM versus Xpert alone was 830 USD/YLS in South Africa and 440 USD /YLS in Malawi [36]. Our findings were consistent to these prior studies that addition of urine-based test to molecular test was the preferred cost-effective strategy compared to molecular tests alone. The present study further generated cost-effectiveness evidence of Xpert Ultra with FujiLAM to be preferred over Xpert alone or Xpert plus AlereLAM assay.

The main driving reason for the cost-effectiveness of FujiLAM +Xpert Ultra was the higher DALYs averted as a results of enhanced early treatment likelihood and reduced late treatment-associated mortality. When these two tests are applied concurrently, the overall sensitivity is higher than either test alone, and therefore reduces false negative results and thus missed or late diagnosed cases. This improvement in diagnosis accuracy can be translated into reduced mortality and DALYs. The addition of urine-based FujiLAM assay (USD 8 per test) to standard sputum-based Xpert Ultra only increased the total direct medical cost modestly by USD4.4 when compared with Xpert Ultra. The cost of adding FujiLAM was offset by the improved detection rate of TBD, thus reduced the resource utilization for clinical judgement-based diagnosis.

Overall, the total cost (model outcome) difference between standard tests with a LAM assay versus standard tests alone was lower than the difference in testing costs (model input) between the single-test and two-test strategies. This was because the standard test plus a LAM assay have enhanced the testing sensitivity (with fewer false negatives) and reduced both the late treatment cases and late treatment-associated mortality (thus lowered the total cost of TBD). The addition of a LAM assay to the standard test therefore reduced cost differences by improving the early detection rate of TBD (and averted the cost associated with late treatment).

Treatment guided by standard sputum-based molecular diagnostic test with urine-based LAM assay has demonstrated association with reduced mortality [11]. The present study provided a model-based framework to translate the clinical findings to assess the cost-effectiveness of adding an adjunctive assay to the standard diagnostic. The model developed in this study is readily to be adopted by other settings (high, intermediate or low burden). By utilizing the region and health system-specific clinical and economic model inputs, health policymakers and clinicians generate health economic evidence to inform the decision on resource allocation for TB testing strategy in HIV-infected patients. The present framework also allows update of model outputs by adopting the sensitivity and specificity of new TB assay in future.

There are several limitations to our study. First, the base-case results were subject to uncertainties in model input parameters. Rigorous sensitivity analyses of all model parameters were therefore performed to examine the robustness of base-case results. The present decision-tree model, simplified the diagnostic algorithm and TBD treatment outcomes, might not fully capture the complexities of TB diagnosis and treatment. The cost analysis was conducted from the perspective of healthcare provider perspective to include direct medical cost, and indirect costs (such as loss of productivity, patient costs, and non–health system costs) were not considered. The cost-effectiveness results might therefore underestimate the health economic benefits generated by the preferred cost-effective strategy. The impact of averted TB transmission from rapid TB diagnostics and treatment initiation was not included, which would be expected to further enhance the health benefits and cost-effectiveness of the preferred option at the community level. Finally, our analysis accounted for false-positive test results in terms of costs of unnecessary anti-TB treatment but did not account for potential life-threatening events resulted from unnecessary anti-TB treatment.

## Conclusion

In conclusion, standard sputum-based TB diagnostic Xpert Ultra with urine-based FujiLAM for TBD testing in HIV-infected individuals appears to be the preferred cost-effective strategy from the perspective of the health service provider of South Africa.

## Supporting information

S1 Table Model inputs of sensitivity and specificity for subgroup analysis. (DOCX)

S2 Table Base-case results of un-dominated strategies in different HIV-infected patient statuses and CD4 cell count levels. (DOCX)
