# Supplementary material for: Standard diagnostics with and without urine-based lipoarabinomannan testing for tuberculosis disease in HIV-infected patients in a high-burden setting–A cost-effectiveness analysis
Source: PLoS One. 2023 Jul 14;18(7):e0288605. doi: 10.1371/journal.pone.0288605 (PMC10348570; doi:10.1371/journal.pone.0288605)
Supplement: S1 Table — (DOCX) [file pone.0288605.s001.docx]

**Table S1****. Model inputs of sensitivity and specificity for subgroup analysis** [1]

| Characteristics of the patient | | AlereLAM | | FujiLAM | |
| --- | --- | --- | --- | --- | --- |
|  |  | Sensitivity | Specificity | Sensitivity | Specificity |
| Patient status | Inpatient | 42.30% | 95.00% | 70.40% | 90.80% |
|  | Outpatient | 27.90% | 95.40% | 70.60% | 90.40% |
| CD4 cell count | ≤ 100 cells/μl | 56.00% | 93.60% | 87.10% | 80.50% |
|  | 101–200 cells/μl | 25.30% | 96.70% | 62.70% | 95.00% |
|  | > 200 cells/μl | 10.90% | 97.60% | 43.90% | 97.00% |

1. Broger T, Nicol MP, Szekely R, Bjerrum S, Sossen B, Schutz C, et al. Diagnostic accuracy of a novel tuberculosis point-of-care urine lipoarabinomannan assay for people living with HIV: a meta-analysis of individual in-and outpatient data. PLoS medicine. 2020;17(5):e1003113.
