# Supplementary material for: Standard diagnostics with and without urine-based lipoarabinomannan testing for tuberculosis disease in HIV-infected patients in a high-burden setting–A cost-effectiveness analysis
Source: PLoS One. 2023 Jul 14;18(7):e0288605. doi: 10.1371/journal.pone.0288605 (PMC10348570; doi:10.1371/journal.pone.0288605)
Supplement: S2 Table — (DOCX) [file pone.0288605.s002.docx]

**Table S2. Base-case results of un-dominated strategies in different HIV-infected patient statuses and CD4 cell count levels.**

| Characteristics of the patient | | Testing strategy | Total direct cost (USD) | Incremental cost | DALYs | DALY averted | ICER (USD/  DALY) |
| --- | --- | --- | --- | --- | --- | --- | --- |
| Patient status | Inpatient | SSM | 1981.1 | - | 0.6869 | - | - |
|  |  | Xpert Ultra | 1984.3 | 3.2 | 0.6421 | 0.0448 | 71.4 |
|  |  | Xpert Ultra+ AlereLAM | 1986.1 | 1.8 | 0.6382 | 0.0039 | 461.5 |
|  |  | Xpert Ultra+ FujiLAM | 1988.7 | 2.6 | 0.6356 | 0.0026 | 1000* |
|  | Outpatient | SSM | 691.0 | - | 0.6869 | - | - |
|  |  | Xpert Ultra | 694.2 | 3.2 | 0.6421 | 0.0448 | 71.4 |
|  |  | Xpert Ultra+ FujiLAM | 698.6 | 4.4 | 0.6356 | 0.0065 | 676* |
| CD4 count | ≤ 100 cells/μl | SSM | 691.0 |  | 0.6869 |  | - |
|  |  | Xpert Ultra | 694.2 | 3.2 | 0.6421 | 0.0448 | 71.4 |
|  |  | Xpert Ultra+ AlereLAM | 695.4 | 1.2 | 0.6370 | 0.0051 | 235.3 |
|  |  | Xpert Ultra+ FujiLAM | 697.5 | 2.1 | 0.6341 | 0.0029 | 724.1* |
|  | 101–200 cells/μl | SSM | 691.0 | - | 0.6869 | - | - |
|  |  | Xpert Ultra | 694.2 | 3.2 | 0.6421 | 0.0448 | 71.4 |
|  |  | Xpert Ultra+ FujiLAM | 699.1 | 4.9 | 0.6364 | 0.0043 | 1139.5* |
|  | > 200 cells/μl | SSM | 691.0 | - | 0.6869 | - | - |
|  |  | Xpert Ultra | 694.2 | 3.2 | 0.6421 | 0.0448 | 71.4 |
|  |  | Xpert Ultra+ FujiLAM | 700.0 | 5.8 | 0.6381 | 0.0040 | 1,450* |

ICER= incremental cost/DALY averted

DALY: disability-adjusted life years; ICER: incremental cost-effectiveness ratio; SSM: sputum smear microscope

* FujiLAM +Xpert Ultra accepted as cost-effective with lowest DALYs and ICER less than willingness-to-pay threshold 7,055 USD/DALY
